# Metabolic features in plasma and urine of obese children and their association with MAFLD risk

**DOI:** 10.3389/fnut.2026.1701999

**Published:** 2026-04-24

**Authors:** Shuang Hu, Wu Yan, Su Wu, Qianqi Liu, Xiaonan Li

**Affiliations:** 1 Department of Children Health Care, Children's Hospital of Nanjing Medical University, Nanjing, China; 2 Department of Endocrinology, Children's Hospital of Nanjing Medical University, Nanjing, China

**Keywords:** LC–MS, MAFLD, metabolomics, obesity, restricted cubic spline

## Abstract

**Objective:**

This study aimed to identify metabolic features in obese children that are associated with metabolic dysfunction-associated fatty liver disease (MAFLD), to explore the dose–response relationship between these metabolites and MAFLD, and to identify any differences depending on sex of the child.

**Methods:**

Obese children recruited from the outpatient clinic were categorized into obese with MAFLD (OB-MAFLD) and obese without MAFLD (OB-CON) groups via B-ultrasound. Anthropometric and laboratory examinations were conducted. Metabolomic analysis was performed using liquid chromatography–tandem mass spectrometry. Principal component analysis (PCA) and orthogonal partial least squares discriminant analysis (OPLS-DA) were employed to identify differential metabolites. Logistic regression models were utilized to evaluate the associations between these metabolites and MAFLD risk, with gender-stratified analyses performed. Restricted cubic spline (RCS) regressions were applied to explore the dose–response relationship between these metabolites and MAFLD.

**Results:**

A total of 160 obese children aged 7–14 years were included, among whom 125 were assigned to the OB-MAFLD group and 35 to the OB-CON group. Four differential metabolites were identified: alanine and proline in plasma and hippuric acid and palmitic acid in urine. Alanine and proline were independent risk factors for the development of MAFLD in obese children, while hippuric acid and palmitic acid were protective factors against MAFLD. After stratification by sex, alanine was specifically associated with MAFLD in obese boys, palmitic acid with MAFLD in obese girls, and hippuric acid with MAFLD in both sexes; in contrast, proline showed no significant association with either group. RCS analysis showed that the associations between MAFLD and all four metabolites followed linear patterns (all *p* > 0.05 for-nonlinearity).

**Conclusion:**

Plasma and urinary metabolite alterations in obese children may confer either susceptibility to or protection against MAFLD development, with distinct sex-specific patterns. These metabolites may serve as quantitative biomarkers and therapeutic targets, advancing precision intervention strategies for obesity-associated MAFLD.

## Highlights

- There were differential metabolites associated with MAFLD in obese children.
- The differential metabolites alanine and palmitic acid exhibit sex-specific effects.
- Alanine and proline are risk factors but hippuric acid and palmitic acid are protective factors.

## 1 Introduction

Pediatric metabolic dysfunction-associated fatty liver disease (MAFLD) is a clinic pathological syndrome characterized by chronic hepatic steatosis in children and adolescents (1). Epidemiological evidence suggests that the global prevalence of pediatric MAFLD parallels the prevalence of obesity, indicating an etiological link between these two metabolic diseases (2). The global prevalence of MAFLD in children is estimated to be approximately 13% (15% in boys and 10% in girls), with higher rates observed in obese populations, reaching up to 47% (54% in boys and 39% in girls) (3).

Risk factors for pediatric MAFLD include diet, adiposity, genetics, microbiome and Gut-Liver axis, developmental programming, puberty, sex hormones, sleep, hypoxia, and environmental exposures (1). Nowadays, growing evidence supports the role of metabolites such as branched-chain amino acids (BCAAs), aromatic amino acids, and other lipidomic profiles in hepatic insulin resistance, hepatic inflammation and/or fibrosis (4–6). These findings may suggest metabolic alterations associated with the onset or progression of MAFLD.

Recent metabolomic studies have identified distinct metabolic signatures associated with pediatric MAFLD, highlighting the potential role of amino acids, lipids, and other metabolites in its pathogenesis. For instance, elevated levels of BCAAs and certain aromatic amino acids have been reported in obese adolescents with MAFLD, correlating with insulin resistance and hepatic fat content (7–9). Similarly, altered lipid metabolism, including increased triglycerides and diglycerides, has been observed in pediatric MAFLD patients, suggesting a link between dysregulated lipid pathways and liver steatosis (10). Additionally, metabolomic profiling of saliva and urine has revealed unique signatures in children with obesity-related MAFLD and metabolic syndrome, indicating the potential for non-invasive biomarkers (11, 12).

These findings underscore the importance of exploring metabolic pathways to better understand MAFLD and develop targeted diagnostic and therapeutic strategies. However, all these studies adopt traditional linear analyses, which assume a simple positive or negative correlation between metabolite levels and MAFLD risk, but fail to reveal the dose-effect relationship. Gender differences in socio-cultural characteristics in pediatric MAFLD, such as dietary choices and exercise behavior, should also be considered for disease risk assessment and precise treatment. There is a growing body of evidence indicating gender dimorphism in pediatric MAFLD, with distinct differences between boys and girls in terms of epidemiology and clinical outcomes (3, 13, 14). Clinical observational studies have confirmed a higher prevalence of MAFLD in obese boys compared to obese girls, regardless of age and Tanner stage (13). Metabolomic profiling study in obese adolescents identified significant sex differences in BCAA-related biomarkers associated with insulin resistance. Their findings revealed that specific BCAA metabolites were elevated in insulin-resistant adolescents, particularly in boys, and correlated with markers of metabolic dysfunction (9). Therefore, it is necessary to explore the relationship between metabolites and the risk of pediatric MAFLD stratified by sex.

In this study, we aimed to identify metabolic features that are associated with MAFLD in the plasma and urine of obese children, to explore the dose–response relationship between these metabolites and MAFLD, and to discover any sex-specific differences. This findings could provide potential biomarkers that can lead to improved treatment options for sex-specific obesity-related MAFLD.

## 2 Methods

### 2.1 Study population

This cross-sectional study included obese children and adolescents aged 7–14 years who were treated in the Department of Child Health Care at the Children’s Hospital of Nanjing Medical University and underwent metabolic assessment. We excluded participants with missing anthropometric, laboratory and image data, any systemic or organic diseases, chronic liver diseases of known etiology; or the use of antihypertensive, antidiabetic, lipid-lowering, uric acid-lowering medications, or hepatotoxic agents. The research was granted approval by the Ethics Committee of Children’s Hospital of Nanjing Medical University (Approval No. 201603004-1).

### 2.2 Anthropometric assessment

Comprehensive physical examinations were conducted for all study subjects. Anthropometric measurements included height, weight, and waist circumference (WC). Height and weight were measured using an electronic scale with participants wearing light clothing without shoes, to the nearest 0.1 cm and 0.1 kg, respectively. Body mass index (BMI) was calculated by dividing weight (kg) by height square (m^2^) and then converted to *Z* score of BMI (BMI-Z), as it corrects for variations in age and sex in children and allows for pooling of data. BMI-Z ≥ 2 indicates obesity. Waist circumference was measured at the level of the umbilicus to the nearest 0.1 cm at the end of gentle expiration. The waist-to-height ratio (WHtR) was calculated by dividing the WC (cm) by height (cm).

### 2.3 Diagnosis of MAFLD

The diagnosis of MAFLD was informed by the Chinese experts consensus on the diagnosis and treatment of MAFLD in children, as published by the Chinese Society of Pediatric Endocrinology and Metabolism (15). MAFLD is identified in children under the age of 18 who meet the following criteria: (1) Chronic hepatic steatosis which is not caused by infections, genetic disorders, alcohol intake, medications, or malnutrition; (2) Elevation of alanine aminotransferase (ALT) was greater than 60 U/L and lasted for more than 3 months; (3) Liver B-ultrasound findings meet the diagnostic criteria for diffuse fatty liver.

### 2.4 Laboratory examination

Following a 12-h overnight fast, venous blood (3 mL) and midstream urine were collected from each participant in the morning. Sterile tubes were used to collect blood and urine samples. Subsequently, the blood and urine samples were centrifuged for 10 min at 3,000 rpm and room temperature. The supernatant obtained was then removed and stored at −80 °C.

### 2.5 Metabolomics

Blood and urine samples collected from these participants were used to assess metabolic differences between obese children with and without MAFLD using untargeted metabolomics.

Liquid chromatography–tandem mass spectrometry (LC–MS/MS) technique was used for the analysis of acylcarnitine in dried blood filters. Initially, venous blood was drawn onto a filter paper sheet with a diameter of about 10 mm, air-dried at room temperature, and stored at −20 °C until analysis. A 3 mm dried blood filter paper was then placed into a 96-well polypropylene plate, and 100 μL of methanol containing an internal standard was added into each well. The plate was left at room temperature for 20 min to allow for extraction, followed by centrifugation into another 96-well polypropylene plate. The extract was then dried under nitrogen at 55 °C. Subsequently, 60 μL of hydrochloric acid (3 mol/L) in n-butyl alcohol was added, covered with a Teflon membrane, and incubated at 65 °C for 15 min to derivatise acylcarnitines to their butyl esters. The plate was dried again at 55 °C, and 100 μL of 80% acetonitrile was added. After sealing with an aluminium membrane, the samples were ready for analysis. Quantitative analysis was performed by computer software (Chem View b5, Bio Applied Systems, USA), which automatically calculated the concentration of acylcarnitines in the samples based on the ion peak intensities of various butylated acylcarnitines and their isotopic internal standards with known concentrations.

Organic acids in urine were determined by gas-chromatography mass spectrometry (GC-MS). Dry urine paper was used for adsorption, removed once saturated, and air-dried naturally before being sealed in a bag and stored at −20 °C for analysis. To remove urea, 20 mL of urease was added to an equivalent of 0.2 mg creatinine in urine, mixed, and incubated at 37 °C for 30 min. After adding 40 μL of the internal standard solution and fixing to 2 mL with ultrapure water, 1,000 μL of 5% ammonium hydroxyl hydrochloride and 400 μL of 5 mol/L sodium hydroxide were added sequentially, mixed, and left at room temperature for 60 min. Organic acids with hydroxyl groups were oximised to produce ketone bodies, followed by the addition of 550 μL of hydrochloric acid, thorough mixing, and PH adjustment to 1–3 to terminate the oximisation reaction. The sample was extracted twice with ethyl acetate, the supernatants were combined, and dried under nitrogen at 60 °C. Finally, the supernatant was derivatised with BSTFA-TMCS (99:1) at 70 °C for 30 min before analysis. Quantification was based on retention time and characteristic ions, and the ratio of the peak area of the product to that of the internal standard used for quantification. GC–MS solution (v2.72) data acquisition software was utilized for data processing, yielding the final semi-quantitative results.

### 2.6 Statistical analysis

The Kolmogorov–Smirnov tests were employed to assess the normality of the quantitative data. Data that were normally are expressed as mean ± SD, with group comparisons made using the Independent sample *t*-test. Data that followed a skewed distribution are represented by median (P_25_, P_75_), and compared using Mann–Whitney *U* test. Qualitative data are expressed as frequency (%), and *chi-square* test was used for comparison.

After the exclusion of metabolites with a detection rate less than 80% (16), the remaining missing values were handled by MetImp (17, 18). PCA was performed to gain an initial visual representation of the differences in metabolite levels, and OPLS-DA model was used to confirm the differentiating metabolites under different sexes separately between obese children with and without MAFLD. The criteria of the differential metabolites were variable importance in the projection (VIP) ≥ 1 and multiple comparisons using FDR < 0.05 according to the method by Benjamini–Hochberg method. Logistic regressions were used to explore the individual effect of each differential metabolite on MAFLD in obese children, adjusted for age, sex, and BMI-Z.

Furthermore, to determine whether there was a nonlinear dose–response relationship of these metabolites with the risk of MAFLD after multivariable-adjustment, restricted cubic splines (RCS) were fitted, with four knots placed at the 5th, 35th, 65th, and 95th percentiles. A *p*-value <0.05 was considered statistically significant. All analyses were performed with SPSS 22.0 and R. version 4.2.0.

## 3 Results

### 3.1 Basic characteristics of obese children in this study

The study enrolled 160 obese children, aged 7 to 14 years, among whom 125 were assigned to the obese with MAFLD (OB-MAFLD) group and 35 to the obese without MAFLD (OB-CON) group. Notably, age, BMI, BMI-Z and WHtR were significantly higher in the OB-MAFLD group than those in the OB-CON group. Additionally, there was a statistically significant difference in sex distribution between the two groups, with a significantly higher incidence of MAFLD in obese boys compared to obese girls (Table 1).

**Table 1:** Basic characteristics of the participants.

Indicators	Obese without MAFLD (*n* = 35)	Obese with MAFLD (*n* = 125)	*t*/*χ*^2^	*p*
Age (y)	10.29 ± 1.81	10.94 ± 1.66	−1.978	**0.049**
Sex			6.791	**0.009**
Boys	18 (51.4)	93 (74.4)		
Girls	17 (48.6)	32 (25.6)		
BMI	25.80 ± 2.71	29.31 ± 3.69	−5.247	**<0.001**
BMI-Z	2.73 ± 0.50	3.27 ± 0.79	−3.779	**<0.001**
WHtR	0.57 ± 0.05	0.62 ± 0.06	−3.955	**<0.001**

BMI, body mass index; BMI-Z, body mass index *Z*-score; WHtR, waist-to-height ratio. Bold values indicate statistically significant differences (*p* < 0.05).

### 3.2 Discriminating metabolic profiles of MAFLD in obese children

#### 3.2.1 Plasma differential metabolite analysis in obese children

**Figure 1:**
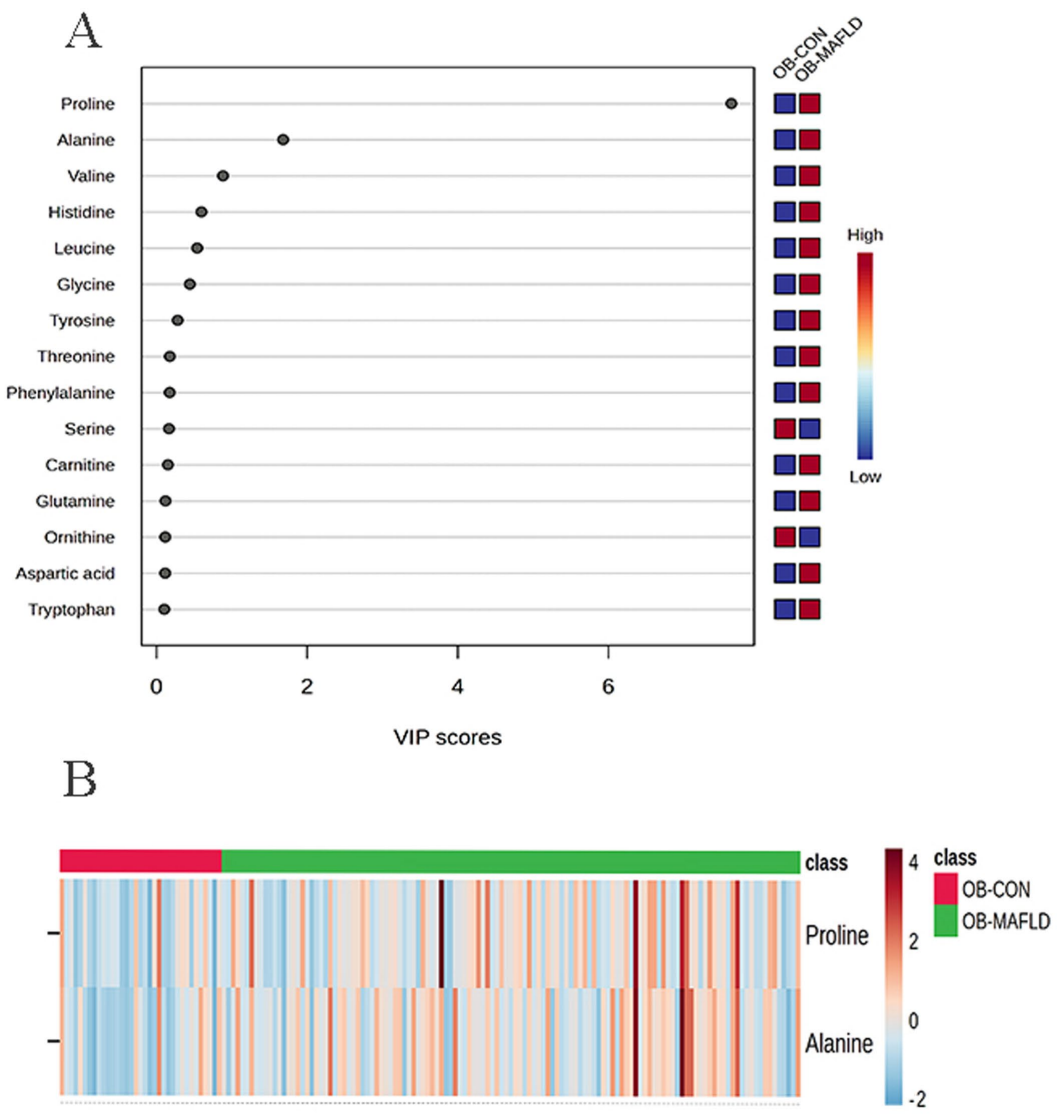
Plasma metabolic profile shift between the obesity control (OB-CON) and the obesity MAFLD (OB-MAFLD) groups. **(A)** VIP scores of the different indicators are screened. Generally, VIP ≥ 1 is considered as an important indicator (the 15 indicators are sorted in descending order). **(B)** Heat map of the altered metabolites (VIP ≥ 1 and FDR < 0.05). The red band indicates upregulation of the metabolite levels, while the blue band indicates downregulation of the metabolite levels in the OB-MAFLD group compared with the OB-CON group.

A total of 106 plasma metabolites were detected, of which 43 were excluded due to a detection rate of less than 80%; 63 metabolites were ultimately included in the analysis. To gain an initial visual representation of the differences in plasma metabolite levels, PCA was performed. The 2D scatter plots (Supplementary Figure S1A) show that the samples of the obesity MAFLD (OB-MAFLD) group and the obesity control (OB-CON) group were almost entirely in the confidence interval of 95%. As shown in Supplementary Figure S1B, the OPLS-DA score plot exhibited substantial overlap, which may be attributed to the fact that these were all samples from obese children. The OPLS-DA model showed poor predictive performance (*R*^2^*Y* = 0.004, *Q*^2^ = 0.004), suggesting no significant metabolic distinction between groups (Supplementary Figure S1C). Although the OPLS-DA model achieved statistical significance in cross-validation (CV-ANOVA *p* = 0.007), the permutation test revealed a positive *Q*^2^ intercept (0.027), indicating model overfitting and lack of reliability (Supplementary Figure S1D). Therefore, univariate statistical methods were employed for differential metabolite identification. Further analysis identified the top 15 metabolites with VIP scores (Figure 1A). Differentially abundant metabolite analysis revealed that alanine exhibited upregulation trends in the OB-MAFLD group compared with the OB-CON group (Figure 1B).

#### 3.2.2 Urine differential metabolite analysis in obese children

**Figure 2:**
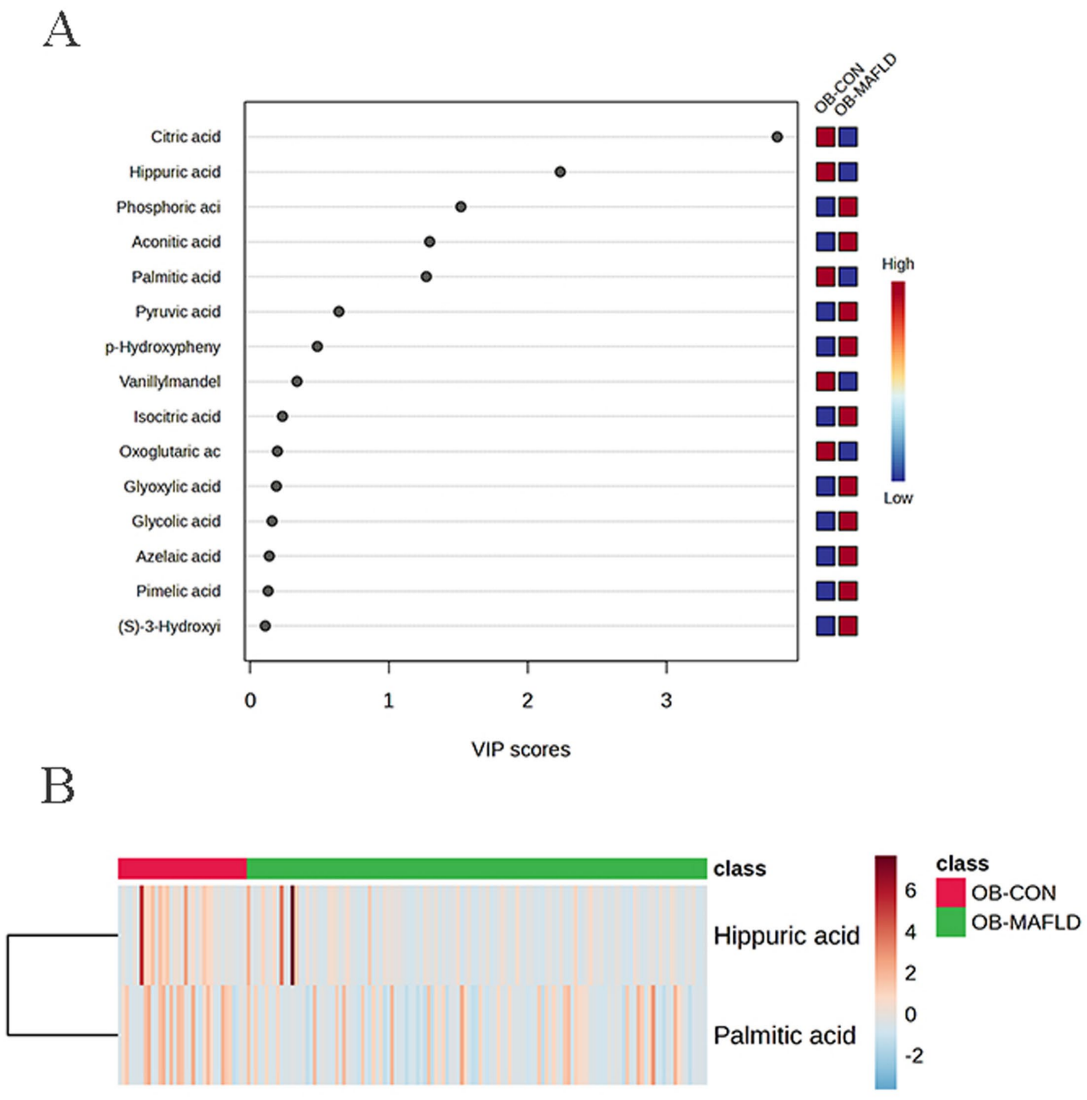
Urine metabolic profile shift between the obesity control (OB-CON) and the obesity MAFLD (OB-MAFLD) groups. **(A)** VIP scores of the different indicators are screened. Generally, VIP ≥ 1 is considered as an important indicator (the 15 indicators are sorted in descending order). **(B)** Heat map of the altered metabolites (VIP ≥ 1 and _FDR_ < 0.05). The red band indicates upregulation of the metabolite levels, while the blue band indicates downregulation of the metabolite levels in the OB-MAFLD group compared with the OB-CON group.

A total of 132 urine metabolites were detected, 26 of metabolites were included in the analysis. The PCA scatter plots (Supplementary Figure S2A) show that the samples of the OB-MAFLD group and the OB-CON group were almost entirely in the confidence interval of 95%.OPLS-DA modeling of urinary metabolites revealed substantial overlap between groups (Supplementary Figure S2B), with poor model performance (*R*^2^*Y* = 0.128, *Q*^2^ = 0.042) (Supplementary Figure S2C). Permutation testing indicated model overfitting (*Q*^2^ intercept = 0.076, despite *p* = 0.001) (Supplementary Figure S2D). Consequently, OPLS-DA was deemed unreliable for class discrimination, and subsequent analyses employed univariate statistical approaches. The top 15 metabolites were identified (Figure 2A). Differentially abundant metabolite analysis revealed that hippuric acid and palmitic acid shown downregulation trend in the OB-MAFLD group compared with the OB-CON group (Figure 2B).

### 3.3 Effect of differential metabolites on the risk of MAFLD in obese children

#### 3.3.1 Effect of plasma differential metabolites on the risk of MAFLD in obese children

We established logistic regression models to assess the effect of each plasma differential metabolite individually on MAFLD in obese children. As shown in Table 2, these two plasma differential metabolites were positively correlated with the occurrence of MAFLD. After adjusting for age, sex, and BMI-Z, alanine and proline were independent risk factors for the development of MAFLD in obese children, with ORs (95%CI) of 1.015 (1.005–1.024) and 1.003 (1.000–1.006), respectively.

**Table 2:** Effects of plasma differential metabolites on MAFLD risk in obese children.

Plasma metabolites	Model 1^†^					
	*β*	SE	Walds	OR	(95%CI)	*p*
Alanine	0.015	0.004	12.020	1.015	1.006–1.023	**0.001**
Proline	0.004	0.001	8.382	1.004	1.001–1.006	**0.004**
	Model 2^†^					
Alanine	0.015	0.005	9.636	1.015	1.005–1.024	**0.002**
Proline	0.003	0.001	4.476	1.003	1.000–1.006	**0.034**

^†^Model 1 were unadjusted models.

Bold values indicate statistically significant differences (*p* < 0.05).

OR, odds ratio; CI, confidence interval.

#### 3.3.2 Effect of urine differential metabolites on the risk of MAFLD in obese children

The individual effects of each urine differential metabolite on MAFLD in obese children were assessed. As shown in Table 3, hippuric acid and palmitic acid were correlated with the occurrence of MAFLD in obese children. Even after adjusting for age, sex, and BMI-Z, these associations remained significant. Hippuric acid and palmitic acid were protective factors for MAFLD, with ORs (95%CI) of 0.974 (0.952–0.996) and 0.978 (0.958–0.998), respectively.

**Table 3:** Effects of urine levels of differential metabolites on NAFLD risk in obese children.

Urine metabolites	Model 1^†^					
	*β*	SE	Walds	OR	(95%CI)	*p*
Palmitic acid	−0.023	0.009	6.335	0.978	0.961–0.995	**0.012**
Hippuric acid	−0.032	0.010	9.141	0.969	0.949–0.989	**0.002**
	Model 2^†^					
Palmitic acid	−0.023	0.010	4.828	0.978	0.958–0.998	**0.028**
Hippuric acid	−0.026	0.012	5.172	0.974	0.952–0.996	**0.023**

^†^Model 1 were unadjusted models.

Bold values indicate statistically significant differences (*p* < 0.05).

OR, odds ratio; CI, confidence interval.

### 3.4 Effect of risk metabolites on MAFLD in obese children stratifying by sex

#### 3.4.1 Effect of plasma risk metabolites on MAFLD in obese children stratifying by sex

After stratifying analyses by gender, sex heterogeneity was found for the effects of alanineon MAFLD among obese children (Table 4). A positive associations of MAFLD with alanine relative level were found in obese boys, but not in obese girls. The effects of proline on MAFLD were similar in obese boys and girls, with no significant correlations observed in either group.

**Table 4:** Effects of plasma risk metabolites on MAFLD risk in obese children stratifying by sex.

Plasma metabolites	Boys (*n* = 111)		Girls (*n* = 49)	
	Adjusted OR (95%CI)^a^	*p*	Adjusted OR (95%CI)^a^	*p*
Alanine	1.020 (1.004–1.037)	**0.016**	1.010 (0.999–1.021)	0.065
Proline	1.004 (0.999–1.008)	0.104	1.002 (0.999–1.005)	0.193

a Adjusted for age, BMI-Z.

Bold values indicate statistically significant differences (*p* < 0.05).

OR, odds ratio; CI, confidence interval.

#### 3.4.2 Effects of urine risk and protective metabolites on MAFLD in obese children stratifying by sex

The effects of hippuric acid on MAFLD were similar in obese boys and girls, with significant negative correlations observed in either sex (Table 5). However, palmitic acid was significantly negatively correlated with MAFLD in obese girls, but not in obese boys.

**Table 5:** Effects of urine risk and protective metabolites on MAFLD in obese children stratifying by sex.

Urine metabolites	Boys		Girls	
	Adjusted OR (95%CI)^a^	*p*	Adjusted OR (95%CI)^a^	*p*
Hippuric acid	0.931 (0.876–0.991)	**0.024**	0.940 (0.890–0.992)	**0.025**
Palmitic acid	0.985 (0.956–1.015)	0.320	0.966 (0.935–0.998)	**0.035**

a Adjusted for age, BMI-Z.

Bold values indicate statistically significant differences (*p* < 0.05).

OR, odds ratio; CI, confidence interval.

### 3.5 Non-linear relationship between metabolites and MAFLD in obese children

#### 3.5.1 Non-linear relationship between plasma risk metabolites and MAFLD in obese children

**Figure 3:**
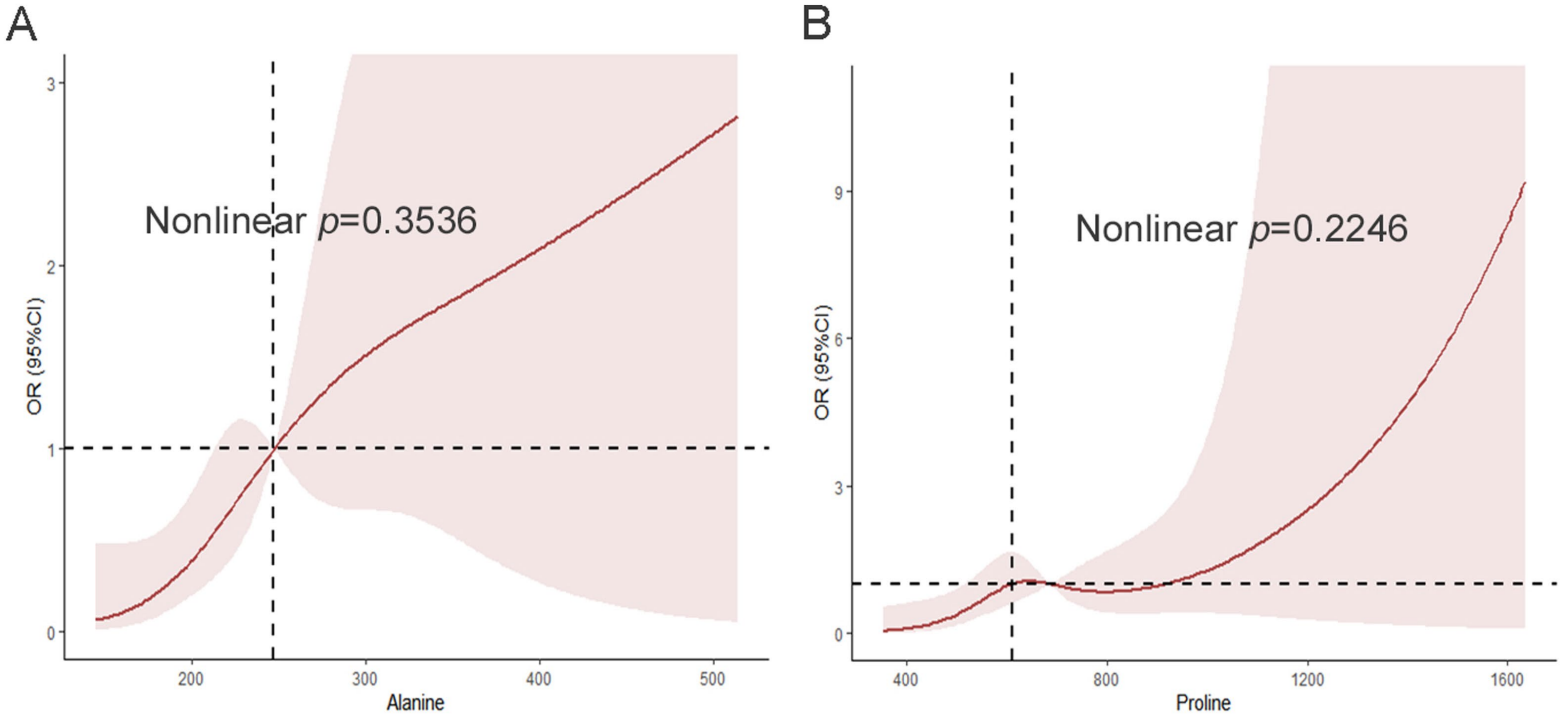
The restricted cubic spline model shows a dose-response relationship between plasma alanine, proline peak intensity, and MAFLD. A) Restricted cubic spline curve between plasma alanine peak intensity and MAFLD; B) Restricted cubic spline curve between plasma proline peak intensity and MAFLD. Adjustments were made according to age, sex, and BMI-Z. The red solid line and the dark red area represent the estimated OR and its corresponding 95% CI, respectively. OR, odds ratio; CI, confidence interval.

To explore the relationship between MAFLD and the two plasma metabolites as independent risk factors, we conducted restricted cubic spline (RCS) regression. As shown in Figure 3A, alanine relative level exhibited a linear positive association (for non-linearity, *p* = 0.3536) with MAFLD risk. In addition, there was a J-shaped trend and a linear positive association (for non-linearity, *p* = 0.2246) between proline relative level and MAFLD risk that was independent of age, sex, and BMI-Z (Figure 3B).

#### 3.5.2 Non-linear relationship between urine protective metabolites and MAFLD in obese children

**Figure 4:**
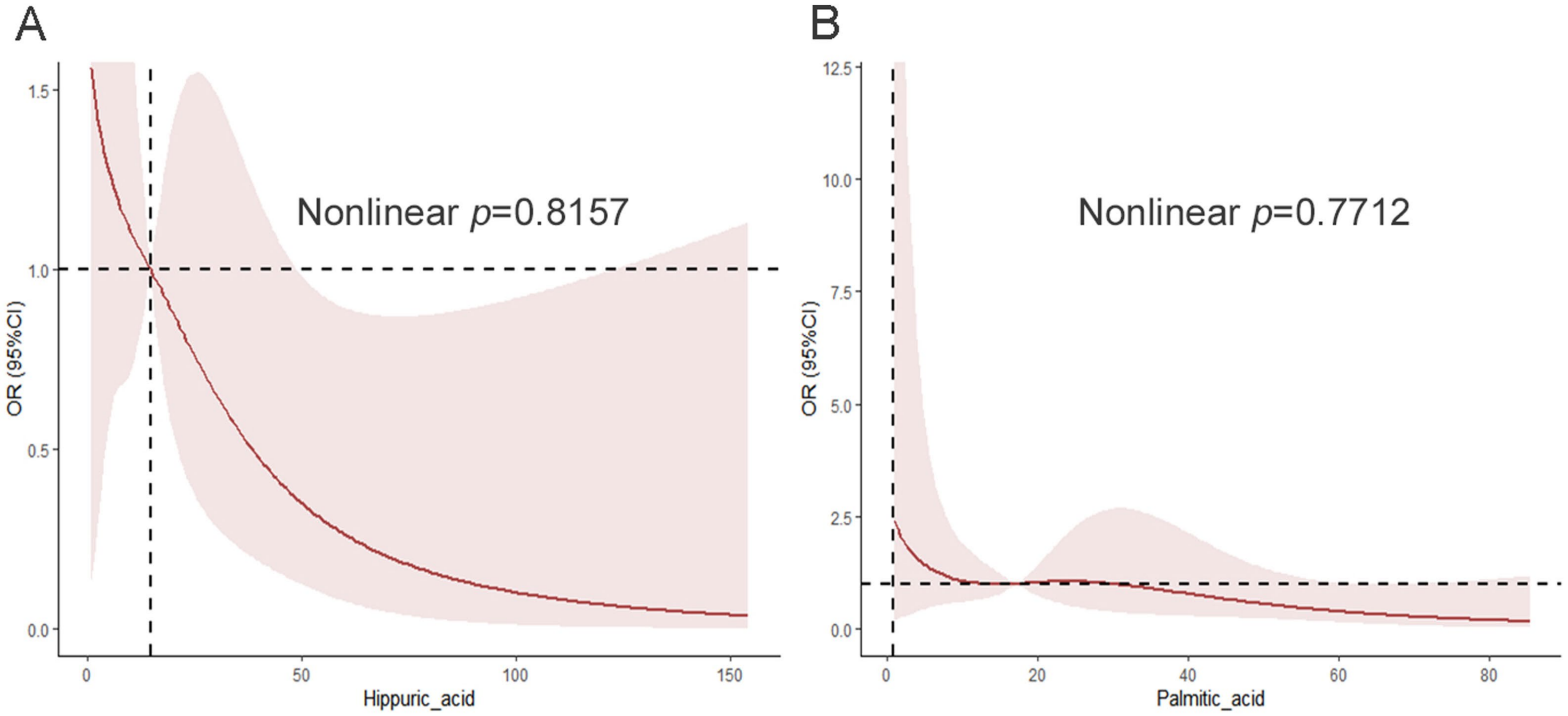
The restricted cubic spline model shows a dose-response relationship between urine hippuric acid, palmitic acid peak intensity, and MAFLD. A) Restricted cubic spline curve between urine hippuric acid peak intensity and MAFLD; B) Restricted cubic spline curve between urine palmitic acid peak intensity and MAFLD. Adjustments were made according to age, sex, and BMI-Z. The red solid line and the dark red area represent the estimated OR and its corresponding 95% CI, respectively. OR, odds ratio; CI, confidence interval.

We also explored the relationship between differential metabolites in urine and MAFLD using RCS. As shown in Figure 4A, the risk of MAFLD gradually decreased with the increase in the relative level of hippuric acid, showing a hyperbolic trend. The risk of MAFLD decreased with increasing relative levels of palmitic acid, showing an approximately linear trend (Figure 4B).

The RCS analysis showed that after adjusting for age, sex, and BMI-Z, the relationships between MAFLD and both hippuric acid and palmitic acid were linear (for *p*-nonlinear = 0.8157, 0.7712), respectively.

## 4 Discussion

This study identified four metabolites (alanine, proline, hippuric acid and palmitic acid) that significantly differed between the MAFLD and non-MAFLD groups in obese children. The metabolites alanine and proline were associated with increased risk of MAFLD among obese children, and hippuric acid and palmitic acid served as protective factors. Moreover, alanine and palmitic acid had sex-specific effects on MAFLD. Specifically, alanine was predominantly linked to the development of MAFLD in obese boys, and palmitic acid was more closely associated with MAFLD in obese girls.

We found that alanine and proline were significant higher in obese children with MAFLD than in those without MAFLD, similar to previous studies (7, 8); these two amino acids could increase the risk of MAFLD in obese children. Elevated plasma alanine exacerbates MAFLD by serving as a gluconeogenic substrate that worsens insulin resistance in the context of selective hepatic insulin resistance (19). Alanine also functions as a direct lipogenic precursor through its conversion to pyruvate and subsequent acetyl-CoA, fueling *de novo* lipogenesis via SREBP-1c activation and promoting hepatic steatosis (20). Proline promotes the progression of MAFLD to fibrosis by serving as the primary amino acid constituent of collagen, directly supporting hepatic stellate cell activation and extracellular matrix deposition in the advanced stages of the disease (21). The accumulation of proline reflects dysregulated collagen turnover and TGF-*β* pathway activation, linking this metabolite specifically to the fibrotic progression characteristic of metabolic dysfunction-associated steatohepatitis (MASH) (21).

We found that hippuric acid is a protective factor for MAFLD in all obese children. Hippuric acid is a glycine conjugate of benzoic acid derived from the catabolism of dietary polyphenols in plant foods by intestinal microflora (22). Population studies have found that hippuric acid is associated with a decrease in liver stiffness (23), and hippurate is related to reduced visceral fat mass (24). Animal studies have shown that high-fat diet-induced NAFLD rats have lower hippuric acid level and impaired antioxidant system and liver function, lipid disorders, abnormal energy, and glucose metabolism (25). Hippuric acid may also adversely affect MAFLD by acting as a uremic toxin, promoting oxidative stress and fibrosis and exacerbating liver disease depending on metabolic context and exposure source (26, 27). These context-dependent effects suggest that the impact of hippuric acid on liver health varies significantly with population, metabolic environment, disease state, and exposure source. Future clinical research should establish large-scale prospective pediatric obesity cohorts to longitudinally validate the causal relationship between hippuric acid levels and MAFLD progression, conduct interventional trials of modulating endogenous hippuric acid through dietary or probiotic approaches, and perform precision stratification analyses based on disease severity and comorbidities to identify populations with differential benefit–risk profiles.

In addition, we observed an inverse linear relationship between palmitic acid and MAFLD risk, which appears contradictory to previous reports in adults (28). This finding may be attributed to the unique metabolic setting of our study, where both cases and controls were obese children with potentially saturated hepatic lipid storage capacity. Under this condition, increased hepatic uptake of palmitic acid for *de novo* lipogenesis may result in decreased circulating levels, reflecting a consumption-driven rather than deficiency-driven phenomenon (29).

We discovered sex dimorphism in the occurrence of MAFLD and related metabolic features: the incidence rate of MAFLD in obese boys was significantly higher than that in girls (13). This may be due to the role of estrogen (30), which can regulate insulin sensitivity, reduce fat synthesis, and promote fat oxidation (31), thereby reducing insulin resistance and indirectly affecting alanine metabolism. Previous studies have shown that the higher incidence rate of insulin resistance in obese boys is associated with the upregulation of alanine, BCAA, and aromatic amino acids like tyrosine and phenylalanine (32). We also found that alanine was a risk factor for MAFLD in obese boys but not in girls. Another reason for the sex dimorphism in NAFLD occurrence in obese children might be The gynoid-type fat distribution with expanded subcutaneous storage capacity in obese girls (33, 34). When saturated, excess palmitic acid overflows from adipose tissue to the liver, reflecting a “lipid storage-overflow” mechanism mediated by estrogen-regulated adipose expandability.

Finally, we employed restricted cubic spline regression to reveal linear dose–response relationships between metabolites and MAFLD, with distinct curve patterns: approximately linear for alanine and palmitic acid, J-shaped for proline, and hyperbolic for hippuric acid. These findings carry significant clinical implications. The linear relationship of alanine with MAFLD aligns with previous studies demonstrating that elevated BCAAs and aromatic amino acids correlate with hepatic insulin resistance in obese adolescents (35). The J-shaped curve of proline suggests potential threshold effects in amino acid metabolism dysregulation, consistent with its role in collagen synthesis and liver fibrosis pathways (36). Notably, the hyperbolic protective pattern of hippuric acid (a gut microbiota-derived metabolite from dietary polyphenols) supports recent evidence that links intrahepatic hippurate levels to improved metabolic dysfunction-associated steatotic liver disease through inhibition of hepatic triglyceride storage (37). This non-linear analysis advances beyond traditional linear models, offering precise biomarker thresholds for pediatric MAFLD risk stratification. Our finding of a linear protective relationship of palmitic acid, which is seemingly contradictory to the findings of studies showing positive associations in adults (27, 33), likely reflects unique metabolic adaptations in obese children with saturated hepatic lipid storage capacity, where decreased circulating levels indicate consumption-driven rather than deficiency-driven phenomena.

Several metabolites that have been previously established as risk factors showed no significant differences or associations with MAFLD in this study, including valine, leucine, isoleucine, and other factors involved in incomplete fatty acid oxidation and lipid peroxidation (7, 8, 10). This discrepancy may be attributed to: (i) control group specificity: obese children without MAFLD may already exhibit altered metabolite levels seen in simple obesity; (ii) disease severity: early-stage MAFLD may not yet show dysregulation of markers associated with advanced disease; (iii) platform limitations: targeted metabolomics did not cover all lipid peroxidation products; and (iv) statistical stringency: FDR correction may have excluded nominally significant metabolites.

Our study has some limitations. The absence of metabolic alterations in the hepatocytes may have affected the interpretation of the data, even though variations in circulating plasma and urine metabolites levels directly represent changes in liver metabolism. For instance, this study was unable to validate the antioxidant effects of important metabolic indicators or the accumulation of free fatty acids in the liver. In addition, the number of obese control group participants was less than that of obese children with MAFLD in this study. However, it has been shown that the comparison results between the two groups were still stable. Moreover, this study lacks detailed auxological assessment, including pubertal staging and growth velocity measurements. Puberty significantly influences metabolic function, and the absence of Tanner staging data limits our ability to account for pubertal effects on the observed sex differences in metabolic biomarkers. Future studies should incorporate comprehensive auxological evaluation to better characterize the interplay between growth, sexual maturation, and metabolic dysfunction. Finally, the small number of obese girls in this study does not exclude possible bias in the results. Future studies needed to further verify the potential mechanisms of the key metabolites and the occurrence of MAFLD, as well as how to coordinate and modify the levels of these metabolites in the obese children.

## 5 Conclusion

This study identified four metabolites associated with MAFLD risk in obese children: alanine and proline as risk factors and hippuric acid and palmitic acid as protective factors. Sex-stratified analysis revealed that alanine specifically affected MAFLD in obese boys, while palmitic acid showed protective effects in obese girls. RCS analysis demonstrated linear dose–response relationships between all metabolites and MAFLD. These findings highlight sex-specific metabolic features in pediatric MAFLD, suggesting potential sex-specific biomarkers for early detection and targeted interventions.

## Data Availability

The raw data supporting the conclusions of this article will be made available by the authors, without undue reservation.
