# Supplementary figures and images for: Metabolic features in plasma and urine of obese children and their association with MAFLD risk
Source: Front Nutr. 2026 Apr 24;13:1701999. doi: 10.3389/fnut.2026.1701999 (PMC13152819; doi:10.3389/fnut.2026.1701999)

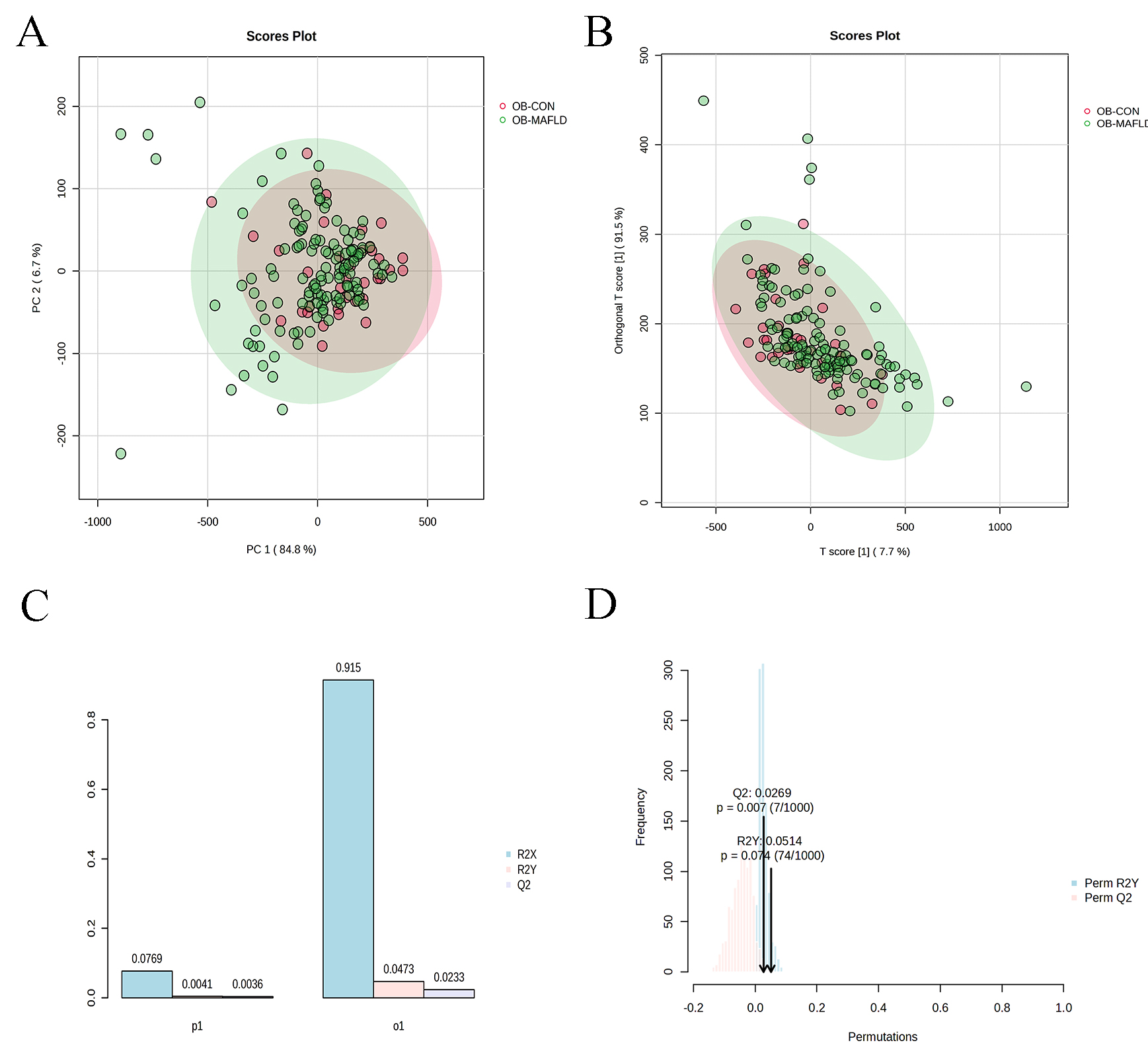

Supplement: Supplementary file 1 [file Image_1.JPEG]

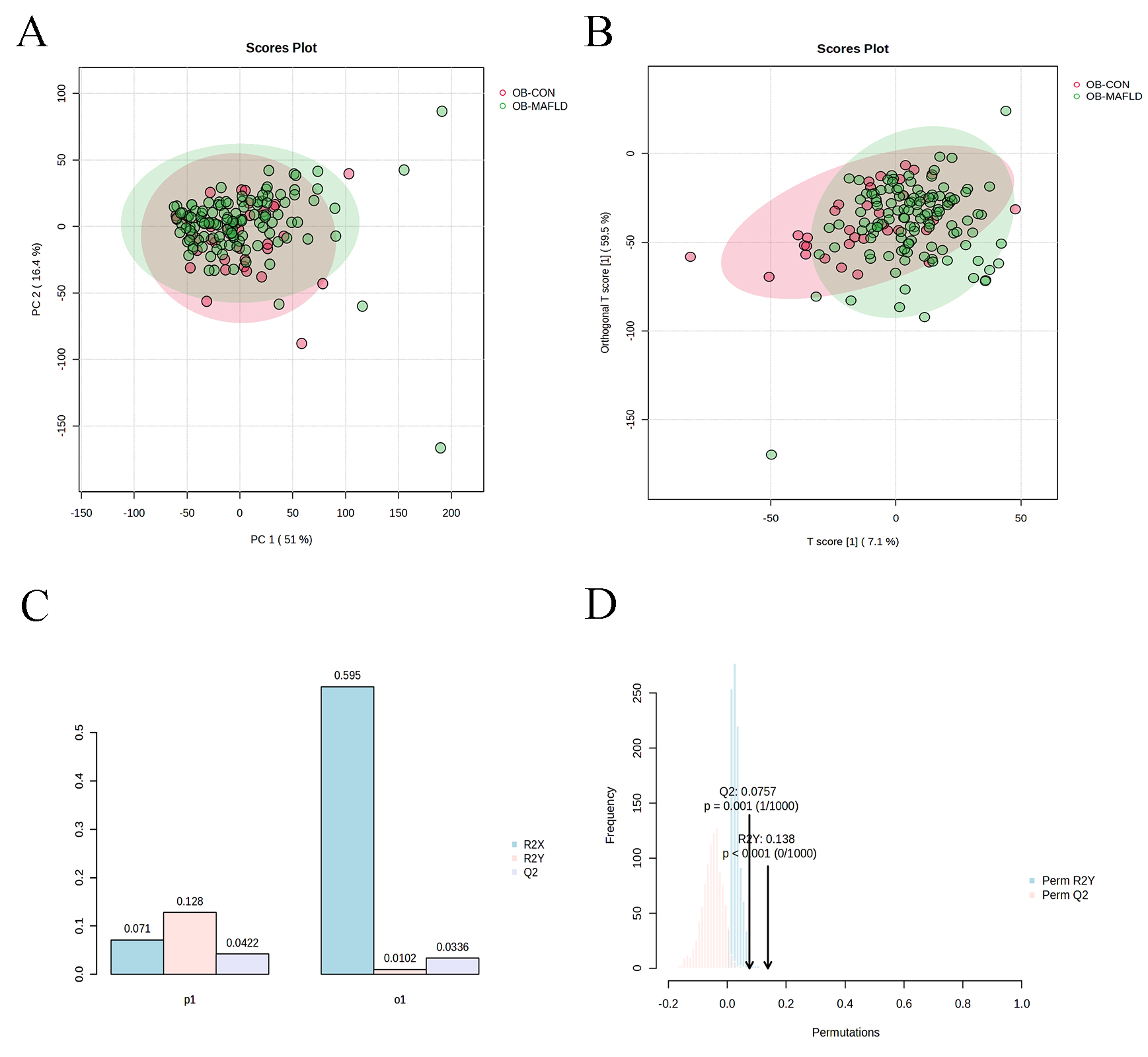

Supplement: Supplementary file 2 [file Image_2.JPEG]
